# A Single Residue Mutation in the Gα_q_ Subunit of the G Protein Complex Causes Blindness in *Drosophila*

**DOI:** 10.1534/g3.117.300340

**Published:** 2017-11-20

**Authors:** Jinguo Cao, Murali K. Bollepalli, Yuhui Hu, Jin Zhang, Qiang Li, Hongmei Li, Hua Chang, Feng Xiao, Roger C. Hardie, Yikang S. Rong, Wen Hu

**Affiliations:** * Department of Medicine, Jinggang Shan University, Ji’an 343009, China; † Department of Physiology, Development and Neuroscience, University of Cambridge, CB2 3DY, United Kingdom; ‡ School of Basic Medical Sciences, Nanchang University, Jiangxi 330031, China; § School of Life Sciences, Institute of Entomology, State Key Laboratory of Biocontrol, Sun Yat-sen University, Guangzhou 510006, China

**Keywords:** phototransduction, photoreceptor, G protein, ERG, Gαq, Gα PLC interaction, light-induced retinal degeneration

## Abstract

Heterotrimeric G proteins play central roles in many signaling pathways, including the phototransduction cascade in animals. However, the degree of involvement of the G protein subunit Gα_q_ is not clear since animals with previously reported strong loss-of-function mutations remain responsive to light stimuli. We recovered a new allele of *Gα_q_* in *Drosophila* that abolishes light response in a conventional electroretinogram assay, and reduces sensitivity in whole-cell recordings of dissociated cells by at least five orders of magnitude. In addition, mutant eyes demonstrate a rapid rate of degeneration in the presence of light. Our new allele is likely the strongest hypomorph described to date. Interestingly, the mutant protein is produced in the eyes but carries a single amino acid change of a conserved hydrophobic residue that has been assigned to the interface of interaction between Gα_q_ and its downstream effector, PLC. Our study has thus uncovered possibly the first point mutation that specifically affects this interaction *in vivo*.

G proteins are essential in the physiological responses to exogenous stimuli. G proteins normally consist of three subunits: Gα, Gβ, and Gγ (Neer 1995; Neves *et al.* 2002). In its inactive state, Gα binds GDP and forms a heterotrimeric complex with Gβ and Gγ. Upon exogenous stimulation, GTP exchange factors, such as G protein-coupled receptors (GPCRs), convert Gα into a GTP-bound state and release Gα from Gβ and Gγ (Siderovski and Willard 2005; Oldham and Hamm 2008; Rosenbaum *et al.* 2009; Campden *et al.* 2015). How Gα activates downstream targets differs according to the types of Gα involved. Gα_s_ and Gα_i_ both act through regulating the level of the secondary messenger cAMP, although in opposite ways (Hildebrandt *et al.* 1983; Sunahara and Taussig 2002; Garcia-Marcos *et al.* 2009). The Gα_q_ subfamily, on the other hand, acts by activating downstream phospholipase C (PLC) (Running Deer *et al.* 1995; Rhee 2001). Activated G protein heightens its GTPase activity by binding to GTPase-activating proteins (*e.g.*, RGS proteins or PLC itself) and converts the GTP-bound state into a GDP-bound one, thus terminating the biological response (Arshavsky and Bownds 1992; Cook *et al.* 2000; Ross and Wilkie 2000; Hollinger and Hepler 2002). Because G proteins are essential for a large number of biological processes and their dysfunction can lead to human diseases such as cancer, the mechanism by which G proteins function has been the subject of intense investigation (Zwaal *et al.* 1996; Ruppel *et al.* 2005; Kelly *et al.* 2006; Shan *et al.* 2006).

The visual system of the fruit fly *Drosophila* has been a fertile ground for studies of G protein. Upon light stimulation, the GPCR rhodopsin is transformed into its activated form, called metarhodopsin, which activates G protein (Lee *et al.* 1990, 1994; Kiselev and Subramaniam 1994; Scott *et al.* 1995). The activated Gα_q_ subunit dissociates from Gβ and Gγ and activates PLC, which in turn generates secondary messengers that ultimately open the TRP and TRPL Ca^++^ channels and results in the depolarization of the photoreceptor cells (Montell and Rubin 1989; Hardie and Minke 1992; Leung *et al.* 2008; Hardie and Franze 2012). Upon termination of the light stimulus, Gα_q_ relocates to the cell membrane, reforms the heterotrimeric complex, and reverts to the inactive GDP-bound conformation. Many aspects of the light response in *Drosophila* can be reliably monitored by the simple electroretinogram (ERG) recording method (Wang *et al.* 2005a; Wang and Montell 2007), which has been widely used to identify mutants that are defective in various aspects of the phototransduction cascade.

Although placed in a central position in the phototransduction cascade, whether the Gα_q_ subunit is essential for transduction has not been firmly established because existing mutants still have some response to light. This may reflect the hypomorphic nature of existing mutations or the fact that *Drosophila* Gα_q_ has numerous splice variants, with different amino acid compositions and different tissue expression patterns (Lee *et al.* 1990; Talluri *et al.* 1995; Alvarez *et al.* 1996; Ratnaparkhi *et al.* 2002). For example, the original *Gα_q_^1^* allele results in the loss of 99% of an eye-specific Gα_q_ protein (quantified by Western blot analysis), yet still retains a substantial ERG response (Scott *et al.* 1995). Moreover, the *Gα_q_^961^* allele with a premature stop codon in the head-specific isoform does not eliminate the ERG response (Hu *et al.* 2012). Moreover, neither mutation causes a rapid light-induced retinal degeneration, whereas other severe loss-of-function mutants of the visual system do.

In this study, we recovered a new *Gα_q_* allele with a single residue change in the most abundant isoform in the adult compound eye. Remarkably, this new allele has a much more severe phenotype than any previously identified *Gα_q_* alleles, yielding an essentially flat ERG response. The mutant eyes also demonstrate a rapid rate of light-induced degeneration. We show that the mutant Gα_q_ protein is still expressed in the eye but is likely nonfunctional. Interestingly, the altered residue lies in a region of Gα_q_ important for its interaction with PLC based on Gα structural studies.

## Materials and Methods

### Drosophila stocks

The genotype of wild-type flies used in our study is *w^1118^*. All flies we used for this study were put into the *w^1118^* background to eliminate the effects of genetic backgrounds. The collection from which our *Gα_q_* allele was recovered was kindly provided by Dr. Yi Rao’s group at Beijing University of China. The mutant stocks of *Gα_q_^1^*, *trp^343^*, and *norpA^P24^* were obtained from Dr. Junhai Han at Southeast University of China. The deficiency stocks and the *gmr-gal4* driver stock (BL8605) were from the Bloomington Stock Center. To avoid light and age-dependent retinal degeneration, flies were reared in standard medium at 25° in the dark and examined when they were 1–2 d old. The three mutations discussed in this study and their location according to Figure 1A of Alvarez *et al.* (1996) are: (1) *Gα_q_^1^*, which is a three amino acid deletion in exon 4A; (2) *Gα_q_^961^*, which is a premature stop in exon 4A; and (3) *Gα_q_^V303D^*, which is in exon 7A.

### Rescuing Gα_q_ phenotypes with transgenes

To generate transgenic flies carrying individual constructs of *UAS-Gα_q_*, *UAS-Gα_q_^V303D^*, or *UAS-Gα_q_^V303I^*, a wild-type cDNA clone of Gα_q_ was changed to carry the V303D or V303I mutations using site-directed mutagenesis. All three cDNA clones were then subcloned into the pUAST-attB vector and introduced into *Drosophila* by phi-C31–mediated transformation. The transgenes were subsequently crossed into the *Gα_q_^V303D^* mutant background and Gα_q_ expression was driven by the eye-specific GMR-Gal4 driver.

### Antibodies

Antibodies used in this study were mouse anti-TRP (83F6) (DSHB), mouse anti-Rh1 (4C5) (DSHB), rabbit anti-Gα_q_ (Calbiochem), rabbit anti-Arr2 (Han *et al.* 2006), rabbit anti-INAD (Wes *et al.* 1999), and anti-PLC (Wang *et al.* 2005b).

### Electrophysiological recording

ERG recordings were performed as previously described (Hu *et al.* 2012). Briefly, 1 or 2-d-old flies were collected, immobilized with strips of tape, and kept in the dark for 5 min before recording. Two glass microelectrodes, filled with Ringer’s solution, were placed on the compound eye and thorax. Flies were stimulated with a Newport light projector for a 5 sec light pulse (2000 Lux). The signal was amplified and recorded using a Warner IE210 Intracellular Electrometer. For each genotype, >10 flies were examined.

### Whole-cell recordings

Whole-cell patch clamp recordings of photoreceptors of dissociated ommatidia from newly eclosed, dark-reared adult flies of either sex were performed as previously described (Hardie *et al.* 2004; Wang *et al.* 2005b). The bath contained (in mM) 120 NaCl, 5 KCl, 10 *N*-Tris-(hydroxymethyl)-methyl-2-amino-ethanesulfonic acid (TES), 4 MgCl_2_, 1.5 CaCl_2_, 25 proline, and 5 alanine (pH 7.15). The intracellular pipette solution (in mM) was 140 K gluconate, 10 TES, 4 Mg-ATP, 2 MgCl_2_, 1 NAD, and 0.4 Na-GTP (pH 7.15).

### Electron microscopy

Electron microscopy (EM) was performed as previously described (Hu *et al.* 2015). Briefly, fly heads were fixed for 2 hr in 0.5% glutaraldehyde, 4% paraformaldehyde, and 0.1 M sodium cacodylate (pH 7.2) at 4°. After three washes with 0.1 M sodium cacodylate, fly heads were stained with 1% osmium tetroxide for 1 hr at room temperature. They were washed three times and stained with uranyl acetate overnight. After a standard ethanol dehydration series, fly heads were rinsed in propylene oxide twice before they were embedded using standard procedures. Thin sections (100 nm) were cut at the top two thirds of retina, collected on Cu support grids, and stained with uranyl acetate for 15 min, followed with 10 min in lead citrate. Micrographs were taken at 120 kV on a JEM-1400 transmission EM.

### Immunostaining

Section staining was carried out as previously described (Tian *et al.* 2013). Briefly, isolated fly heads were fixed for 2 hr at 4° with 4% paraformaldehyde in PBS. The fly heads were dehydrated with acetone and embedded in LR White resin. Cross-sections of 1 μm were made across the top two thirds of retina, collected, and stained with antibodies (Rh1, 1:200; INAD, 1:400; TRP, 1:400). After being washed in PBS, cross-sections were incubated with secondary antibodies and Phalloidin at room temperature for 1 hr. The stained sections were examined under a ZEISS Axio Image A2 microscope.

### Gα_q_ protein translocation assay

Gα_q_ translocation assay was performed as described previously (Frechter *et al.* 2007). Wild-type and mutant flies were each separated into three groups and treated differently. The D group (dark) was kept in the dark for 2 hr before they were killed for Western blotting. The L group (light) was kept in the dark for 2 hr, and then exposed to bright light for 2 hr before being killed. The LD group (light and dark) was kept in the dark for 2 hr, then exposed to bright light for 2 hr, and finally returned to complete darkness for 2 hr. Flies were snap-frozen in liquid nitrogen, and the heads isolated and homogenized in PBS. Pellets and supernatant fractions were separated by centrifuging at 14,000 × *g* for 4 min before subjecting to Western blot analysis.

### Data availability

The research reagents generated in this study are freely available upon request. The authors affirm that all data necessary for confirming the conclusions presented in the article are represented fully within the article.

## Results

### A new Gα_q_ allele with a flat ERG response

**Figure 1:**
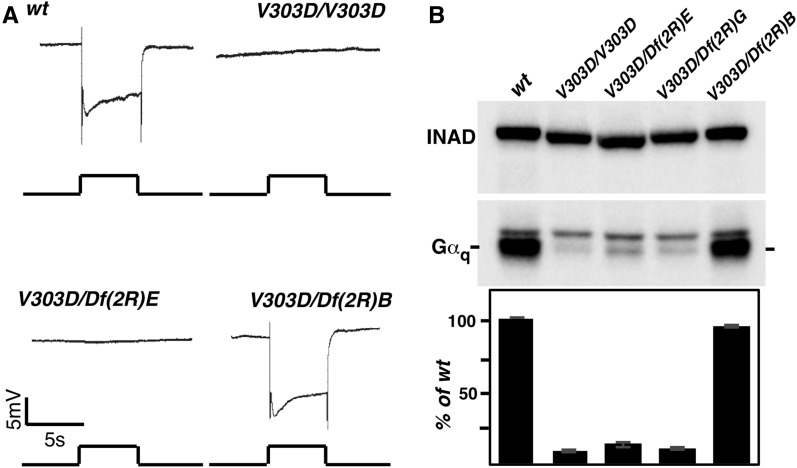
A new *Gα_q_* mutant with a flat ERG response. (A) ERG recording in various genetic backgrounds. Flies that are either homozygous for the *V303D* mutation or *trans*-heterozygous for *V303D* and a chromosomal deficiency uncovering the *Gα_q_* region “*Df*(*2R*)*E*” (abbreviated for *Df*(*2R*)*Exel7121*) show a nearly complete loss of response to light stimulation. However, flies *trans*-heterozygous for *V303D* and a chromosomal deficiency uncovering an adjacent region to *Gα_q_* “*Df*(*2R*)*B*” (abbreviated for *Df*(*2R*)*BSC485*) displayed a normal ERG recoding. For all ERG recordings, event markers represent 5-sec orange light pulses, and scale bar for the vertical axis is 5 mV. (B) The level of Gα_q_ protein in various genetic backgrounds. Western blot was used to detect Gα_q_ protein level in whole exact from fly heads with the indicated genotypes. “*Df*(*2R*)*G*” is the abbreviation for *Df*(*2R*)*Gαq1.3*. In each genotype, the Gα_q_ band is marked and the upper band is nonspecific. INAD was used as a loading control. Quantification of the Western blot results is shown below. The complete genotypes are as follows: *w^1118^* (*wt*); *w^1118^*; *Gα_q_^V303D^* (*V303D*); *w^1118^*; *Gα_q_^V303D^/Df*(*2R*)*Exel7121* (*V303D/Df*(*2R*)*E*); *w^1118^*; *Gα_q_^V303D^/Df*(*2R*)*Gαq1.3* (*V303D/Df*(*2R*)*G*); *w^1118^*; *Gα_q_^V303D^/Df*(*2R*)*BSC485* (*V303D/Df*(*2R*)*B*).

**Figure 2:**
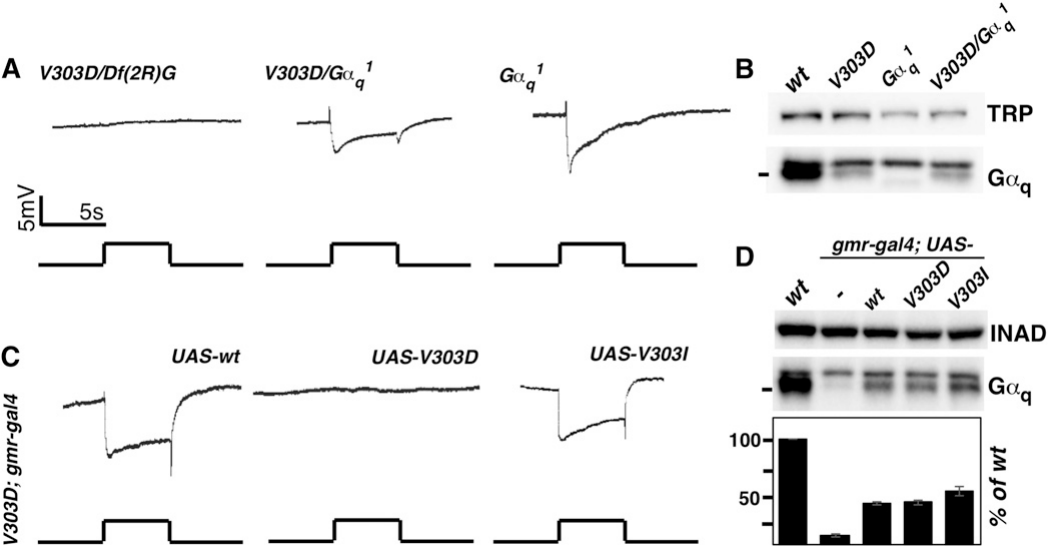
Defective Gα_q_ protein but not the reduction in Gα_q_ level is responsible for the loss of a light response. (A) ERG recordings of Gα_q_ mutants. Flies *trans*-heterozygous for *V303D* and the deficiency *Df*(*2R*)*Gαq1.3* displayed no light response. Mutants either homozygous for the *Gα_q_^1^* mutation or *trans*-heterozygous for *Gα_q_^1^* and *V303D* displayed a substantial response to light. (B) Western blot analyses of Gα_q_ protein level showed that Gα_q_ level is lower in *Gα_q_^1^* mutants than in *V303D* homozygous mutants. TRP serves as a loading control. (C) The ERG recordings of *V303D* mutants expressing different Gα_q_ variants. Flies carrying homozygous *V303D* mutation, a *GMR-Gal4* transgene, and different *UAS-Gα_q_* transgenes were subject to ERG recording. Both the wild-type Gα_q_ and the mammalian mimic V303I transgenes rescued the ERG phenotype. For all ERG traces, event markers represent 5-sec orange light pulses, and scale bars are 5 mV. (D) Western blot measurement of Gα_q_ protein level in rescued lines. Gα_q_ level was restored to 40% of the wild-type level when GMR-Gal4 was used to drive *Gα_q_* expression. INAD served as a loading control. Quantification of the Western blot results is given below. The complete genotypes are as follows: *w^1118^* (*wt*); *w^1118^*; *Gα_q_^V303D^* (*V303D*); *w^1118^*; *Gα_q_^V303D^/Df*(*2R*)*Gαq1.3* (*V303D/Df*(*2R*)*G*); *w^1118^*; *Gα_q_^1^* (*Gα_q_^1^*); *w^1118^*; *Gα_q_^V303D^/Gα_q_^1^* (*V303D/Gα_q_^1^*); *w^111^*^8^; *Gα_q_^V303D^ gmr-Gal4*; *UAS-Gα_q_^+^*; *w^111^*^8^; *Gα_q_^V303D^ gmr-Gal4*; *UAS-Gα_q_^V303D^*; *w^111^*^8^; *Gα_q_^V303D^ gmr-Gal4*; *UAS-Gα_q_^V303I^*.

We have been using the ERG recording method to screen mutagenized *Drosophila* collections to uncover new players in the phototransduction cascade. We recovered a new mutant line with a flat ERG response (Figure 1A and Figure 2A). Genetic mapping based on the loss of a ERG response revealed that the new mutation is uncovered by the chromosomal deficiencies of *Df*(*2R*)*Exel7121* and *Df*(*2R*)*Gα_q_1.3*, which include the *Drosophila Gα_q_* locus. Genomic sequencing identified a single T to A nucleotide change in *Gα_q_*, making it the prime candidate for the responsible gene. This mutation results in a Val to Asp change at residue 303, and the mutant was thus named *Gα_q_^V303D^*, or *V303D* for short. The V303 residue is specific to the *Gα_q_* isoform in the eye.

To confirm that the *V303D* mutation is responsible for the flat ERG response, we introduced a wild-type copy of the *Gα_q_* cDNA driven by the eye-specific GMR promoter into *V303D* homozygotes, or *V303D* *trans*-heterozygotes with a *Gα_q_* deficiency, and was able to rescue the ERG response in both cases (Figure 2C). Therefore, the defective ERG response in our mutant is caused by a defective *Gα_q_* gene. It is worth noting that before our work, only a few genetic backgrounds were shown to produce a flat ERG response: single mutations in the *rdgA* gene that encodes diacylglycerol kinase (Masai *et al.* 1997; Raghu *et al.* 2000) and the *norpA* gene that encodes PLC (McKay *et al.* 1995; Kim *et al.* 2003), or double mutations in the *trp* and *trpl* channels (Leung *et al.* 2000, 2008; Yoon *et al.* 2000). This suggests that the new *Gα_q_* mutation that we identified is likely to be one of the strongest mutations of the phototransduction cascade in *Drosophila*.

### Gα_q_^V303D^ flies undergo rapid retinal degeneration

**Figure 3:**
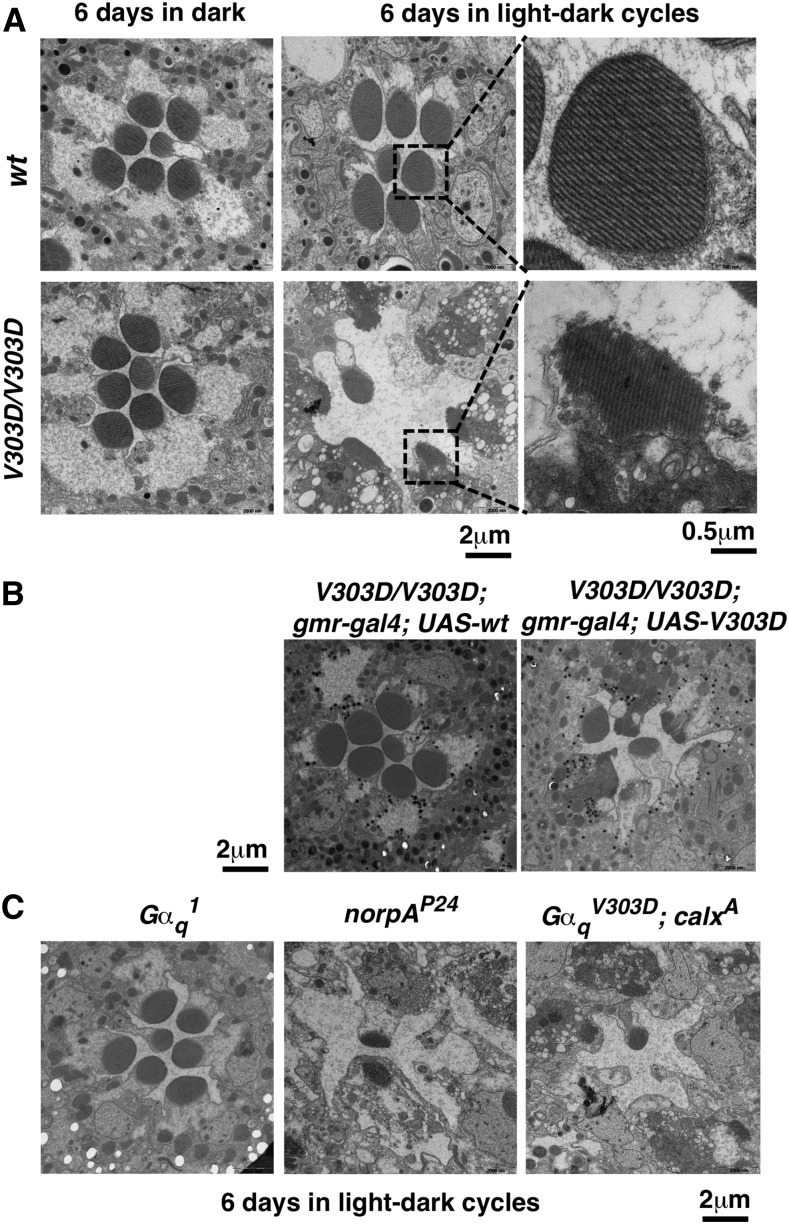
*Gα_q_^V303D^* mutants undergo rapid light-dependent retinal degeneration. (A) Electron microcopy images of an ommatidium from wild-type and *V303D* mutant eyes, with higher magnification images of selected rhabdomeres (highlighted with a square) shown to the right. Flies were raised for 6 d under either constant dark condition or a 12 hr light/12 hr dark cycle. (B) The GMR-driven wild-type *Gα_q_* transgene, but not the *V303D* mutant transgene, rescues visual degeneration of the *V303D* mutant. Scale bars are indicated at the bottom. (C) Retinal degeneration did not happen in similarly dark/light-treated 6-d-old eyes from *Gα_q_^1^*. Fast degeneration of *V303D* eyes is similar to *norpA* mutants, and could not be relieved by a *calx* mutation. The complete genotypes are as follows: *w^1118^* (*wt*); *w^1118^*; *Gα_q_^V303D^* (*V303D*); *w^111^*^8^; *Gα_q_^V303D^ gmr-Gal4*; *UAS-Gα_q_^+^*; *w^111^*^8^; *Gα_q_^V303D^ gmr-Gal4*; *UAS-Gα_q_^V303D^*; *w^1118^*; *Gα_q_^1^*; *w^1118^*; *norpA^P24^*; *w^1118^*; *Gα_q_^V303D^*; *calx^A^*.

Many mutants in the *Drosophila* phototransduction cascade display light-dependent retinal degeneration, including flies with previously identified *Gα_q_* mutants (Hu *et al.* 2012). We raised *Gα_q_^V303D^* adults under either regular light-dark cycles or constant dark conditions, and assayed retinal degeneration using EM. We observed severe degeneration in eyes taken from 6-d-old *Gα_q_^V303D^* mutants raised under light-dark cycles (Figure 3A), but not from those reared in constant dark (Figure 3A). This degree of light-dependent retinal degeneration was more severe than in previously identified *Gα_q_^1^* mutants (Figure 3B). Under similar rearing conditions, *Gα_q_^1^* and *Gα_q_^961^* mutant eyes display visible degeneration only after 21 d posteclosion (Hu *et al.* 2012). As shown in Figure 3B, this degree of fast degeneration in *V303D* mutants resembles that in *norpA* mutants (loss of PLC), suggesting that the phototransduction pathway in the mutants might have terminated before reaching PLC. Importantly, this visual degeneration of *Gα_q_^V303D^* eyes was rescued by the GMR-driven *Gα_q_* transgene (Figure 3B). Interestingly, increasing Ca^++^ concentration with the *calx^A^* mutation was not able to rescue the degeneration phenotype (Figure 3C). Therefore, it is unlikely that a drop in Ca^++^ level in *Gα_q_^V303D^* eyes leads to degeneration by preventing RdgC’s dephosphorylation of M-PPP (Wang *et al.* 2005b).

### Gα_q_^V303D^ encodes a nonfunctional protein

Both the *Gα_q_^1^* and *Gα_q_^961^* alleles previously identified behave as strong loss-of-function alleles (Figure 2A). However, the new *Gα_q_^V303D^* allele lacks a response on a conventional ERG setting, although it does produce a small response with very bright illumination (see Figure 6). Interestingly, *Gα_q_^V303D^*/*Gα_q_^1^* *trans*-heterozygotes behave similarly to *Gα_q_^1^* homozygous mutants (Figure 2A), consistent with *Gα_q_^1^* being a hypomorphic mutation and *V303D* being a functionally null mutant based on ERG recordings. Since the *Gα_q_^961^* mutant is no longer available, we were not able to test its genetic relationship with *V303D*.

Similar with other *Gα_q_* mutants, *V303D* results in a substantial reduction in protein level (∼10% of the wild-type level remaining) as shown by Western blot analyses of total proteins from adult heads (Figure 1B and Figure 2, B and D). However, it is unlikely that this reduction of Gα_q_ protein alone could account for the essentially complete loss of visual capacity in *V303D* mutants, since *Gα_q_^1^* results in a more severe loss of Gα_q_ protein (Figure 2B) yet retains a substantial ERG response (Figure 2A). To provide direct evidence supporting the proposition that the visual defects in *V303D* are at least partly due to the production of a defective Gα_q_ protein, we tested the effect of increasing the level of the V303D mutant protein. As shown in Figure 2D, *GMR*-driven expression of the wild-type Gα_q_ protein, although only reaching ∼40% of the endogenous Gα_q_ level in wild-type eyes, is sufficient to rescue the ERG defect in *V303D* mutants (Figure 2C). On the other hand, when the mutant V303D protein was expressed at a similar level (Figure 2D), it was still unable to rescue the ERG defect (Figure 2C). Therefore, a simple elevation of the Gα_q_^V303D^ protein is insufficient to restore visual function, implying that the V303D protein is itself defective, and that its nonfunctionality might have led to its instability.

### Other components in the phototransduction cascade are normal in Gα_q_^V303D^ mutants

The flat ERG response of *Gα_q_^V303D^* eyes resemble those produced by severe loss-of-function mutations of other components in the phototransduction cascade, such as those in the PLC enzyme (Harris and Stark 1977; McKay *et al.* 1995; Kim *et al.* 2003; LaLonde *et al.* 2005) and the TRP and TRPL channels downstream of the G protein (Leung *et al.* 2000, 2008; Yoon *et al.* 2000; Popescu *et al.* 2006). This suggests that the V303D mutant Gα_q_ is unable to support phototransduction; however, we also considered the possibility that *V303D* is a neomorphic mutation and that the mutant protein indirectly affects the development of photoreceptor cells or the function of other components of the cascade.

We first ruled out that the *V303D* mutation is dominant or semidominant. When V303D was expressed in the wild-type background, using the GMR promoter, we did not observe any discernible visual defect. This was also the case when we raised the relative level of V303D protein further by lowering the wild-type dosage of Gα_q_ with heterozygous *Gα_q_* deficiencies. In other words, eyes hemizygous for *Gα_q_* and expressing *gmr-V303D* are normal under our experimental conditions. Therefore, *V303D* is highly unlikely to be dominant to the wild-type allele.

**Figure 4:**
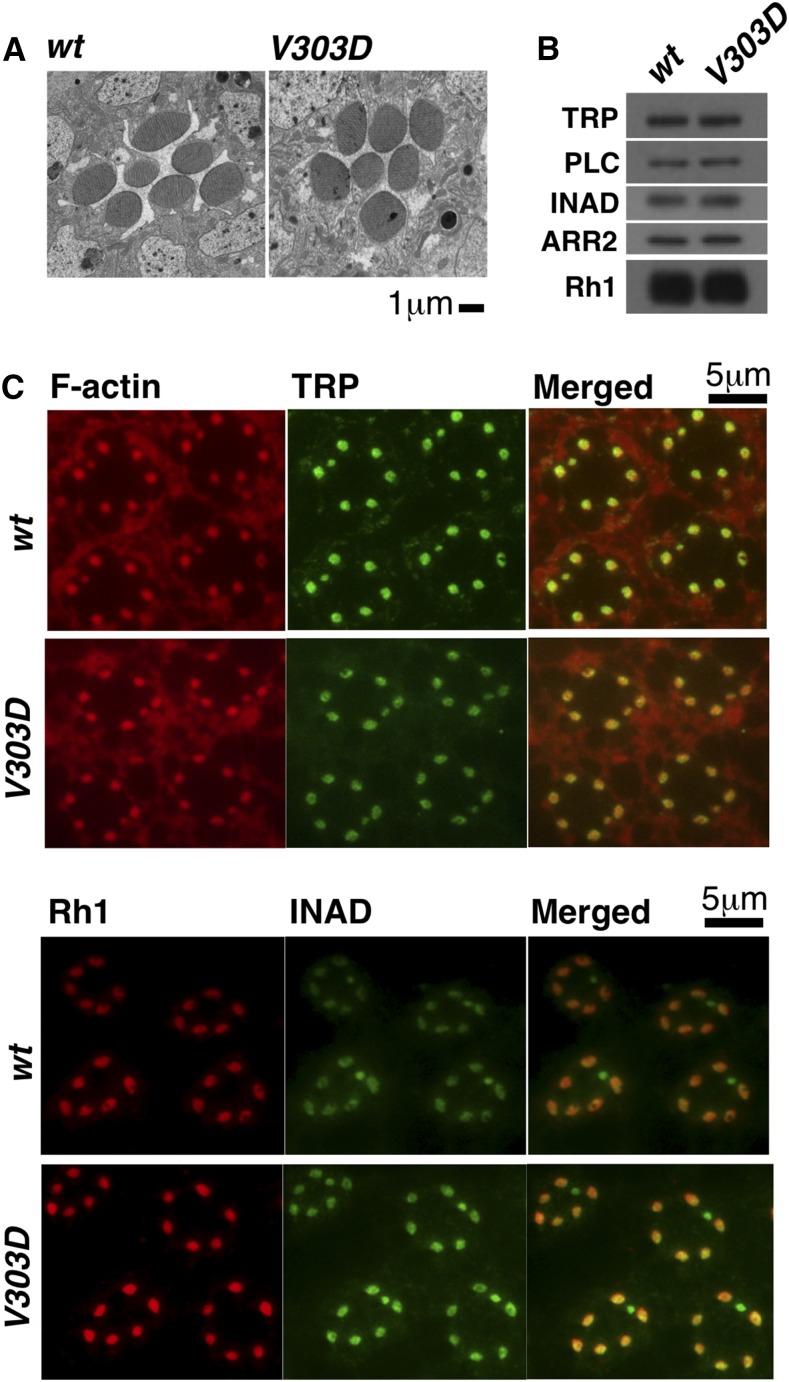
Normal rhabdomere structure and distribution of other visual factors in *Gα_q_^V303D^* mutant. (A) EM images of 1-d-old wild-type and *Gα_q_^V303D^* eyes showing normal rhabdomere structure. (B) Western blot results showing protein levels of phototransduction factors are similar between wild type and *V303D* mutants that were 1 d old. (C) Immunostaining results showing normal distribution of phototransduction factors in *Gα_q_^V303D^* mutant flies. The complete genotypes are as follows: *w^1118^* (*wt*); *w^1118^*; *Gα_q_^V303D^* (*V303D*).

To assess the integrity of other components of the cascade, we checked the structure of photoreceptor cells by EM, the localization of Rh1, TRP, and INAD proteins by immunostaining, and the level of Rh1, TRP, INAD, PLC, and ARR2 proteins by Western blot analysis. To eliminate the secondary effect of degenerated retinal structure suffered by older *V303D* eyes, our analyses were performed on samples taken from 1-d-old adults, when the overall eye structural remains normal (Figure 4A). As shown in Figure 4, B and C, neither the localization nor the expression level of the various protein components of the phototransduction cascade were altered by the *V303D* mutation. Therefore, the mutation affects Gα_q_ specifically.

### The V303D mutation might disrupt the interaction between Gα_q_ and PLC

**Figure 5:**
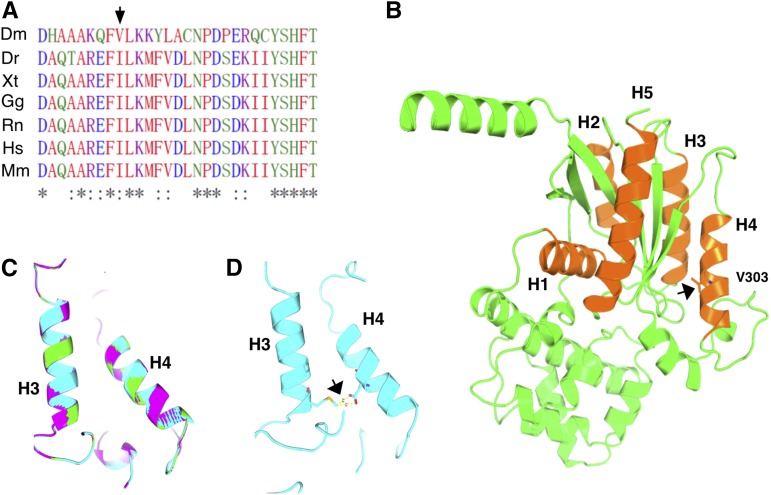
The molecular model of the V303D protein. (A) Alignment of the V303 region in Gα_q_ proteins. The V303 residue is labeled with an arrow. (B) The structure of Gα_q_ modeled over known Gα structures, with the helices (H) involving in interaction with GPCR and PLC labeled in numbers. V303 is situated on helix 4, with its side chains shown and highlighted with an arrow. Helices 3 and 4 participate in interacting with PLC. (C) The predicted structures of helices 3 and 4 in wild type Gα_q_ (green), Gα_q_^V303I^ (purple), and Gα_q_^V303D^ (cyan) proteins are overlaid to highlight a lack of major structural disruption of the V303D mutation. (D) In V303D, the side chain of the D303 mutant residue might participate in hydrogen bonding with M242 on helix 3 as indicated by the arrow. Dm, *Drosophila melanogaster*; Dr, *Danio rerio*; Gg, *Gallus gallus*; Hs, *Homo sapiens*; Mm, *Mus musculus*; Rn, *Rattus norvegicus*; Xt, *Xenopus tropicalis*.

An alignment of the Gα_q_ proteins from various organisms revealed that the V303 residue lies in an important region of Gα_q_ proteins (Figure 5A). Structural analyses of the Gα proteins with regards to its interaction with PLC identified the V303 region as the interface between the two proteins (Noel *et al.* 1993; Lambright *et al.* 1994, 1996; Alvarez *et al.* 1996). Interestingly, Gα_q_ proteins in higher eukaryotes exhibit isoleucine at the position equivalent to the valine residue in the fly protein (Figure 5A), with both being hydrophobic. It is conceivable that the change of a hydrophobic residue to a polar one (D in the *V303D* mutant) can exert a large effect on the interaction between the two proteins. Consistent with this hypothesis, we were able to rescue the visual defects associated with V303D when we expressed a V303I variant of the fly protein (Figure 2C). We modeled the mutant protein using published structures of Gα_q_ proteins. As shown in Figure 5C, neither the V to D nor the V to I change would lead to a dramatic change of the three-dimensional structure of Gα_q_. The V303 residue is situated in helix 4 of Gα (Figure 5B). Interestingly, our structural model predicts that the side chains of a mutant Asp at position 303 would be in close proximity with Met at 242 in helix 3, another part of Gα_q_ important for PLC interaction. The two residues might form hydrogen bonding, potentially affecting the Gα_q_–PLC interaction (Figure 5D).

Therefore, the defect of V303D could simply be that the mutant Gα_q_ protein is unable to interact with and hence activate PLC. We attempted to use immunoprecipitation to investigate Gα_q_-PLC interaction. However, we were unable to detect association even under the wild-type condition. Nevertheless, the above hypothesis predicts that the lack of a photo response is simply due to the inability of the mutant protein to relay the signal, and that the downstream cascade should be functional in *Gα_q_^V303D^* mutant. Our prior results showing normal expression level and localization of other components of the phototransduction cascade is consistent with this hypothesis (Figure 4).

**Figure 6:**
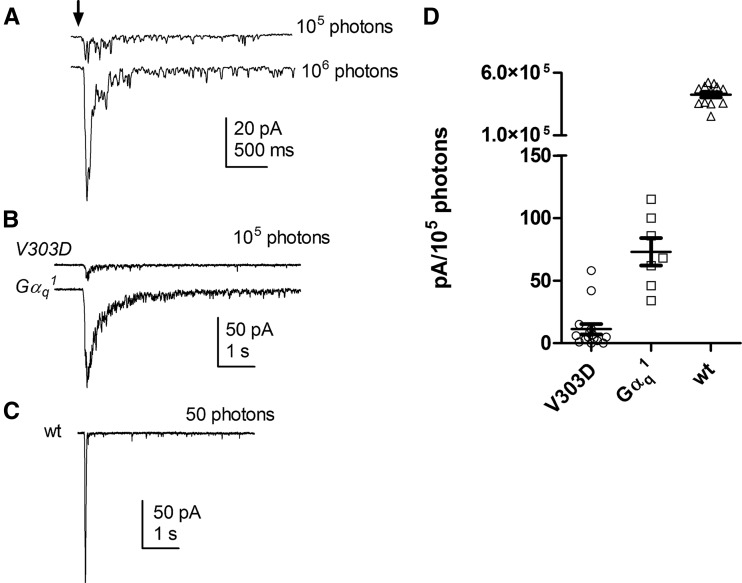
Light responses measured by whole-cell recording. (A) *Gα_q_^V303D^* mutants display greatly reduced responses to 10 msec flashes containing ∼10^5^ and 10^6^ effective photons. (B) *Gα_q_^V303D^* mutant’s response to 100 msec flashes containing 10^5^ photons was greatly reduced when compared with that of *Gα_q_^1^* mutants. (C) A wild-type response is shown. (D) Summary data of peak amplitudes in response to flashes containing 10^5^ photons in *wt* (*n* = 11), *Gα_q_^V303D^* (*n* = 15), and *Gα_q_^1^* (*n* = 7) mutants. The complete genotypes are as follows: *w^1118^* (*wt*); *w^1118^*; *Gα_q_^V303D^* (*V303D*); *w^1118^*; *Gα_q_^1^* (*Gα_q_^1^*).

To gain further evidence that the cascade was otherwise intact, we used whole-cell recording to investigate photoreceptor integrity and whether the function of the TRP channels is normal in the mutant eye. Consistent with our ultrastructural (EM) studies, dissociated ommatidia from *V303D* mutants appeared normal in appearance. Whole-cell recordings showed no sign of constitutive channel activity and cells had capacitances (59.8 ± 2.2 pF; *n* = 15), similar to wild-type and essentially identical to that in *Gα_q_^1^* mutant (58.4 ± 3.1 pF; *n* = 8), indicating that the area of microvillar membrane was unaffected. Interestingly, under whole-cell recording conditions, most *V303D* mutant photoreceptors did display a slight response to very bright light stimuli, but with an ∼10-fold reduced sensitivity compared with the *Gα_q_^1^* mutant (Figure 6). The kinetics and channel noise of these residual response were similar to those in *Gα_q_^1^*, suggesting that downstream components (PLC and TRP/TRPL channels) were functioning normally. Whether these responses were due to minimal residual function of the V303D mutant or an alternative G protein isoform is unclear.

### Impaired long-term adaptation in the V303D mutant

**Figure 7:**
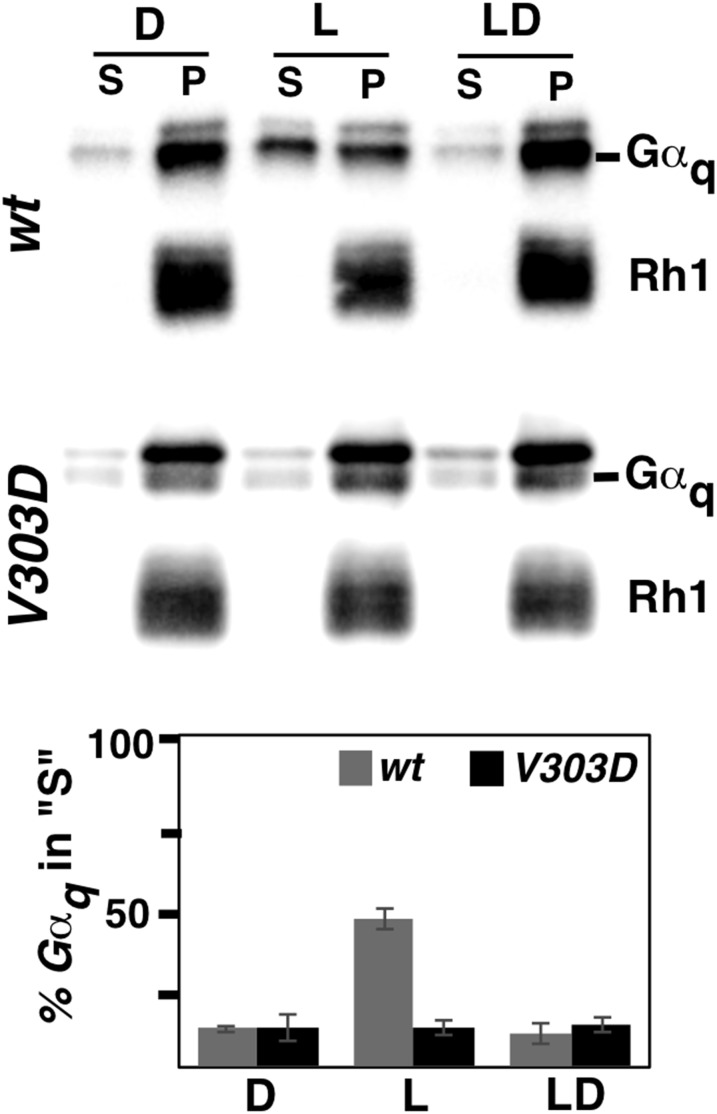
The Gα_q_^V303D^ protein is defective in cytoplasmic translocation induced by constant light stimulation. Wild-type and *V303D* mutant flies were each separated into three groups and treated differently (for treatment details see *Materials and Methods*). Supernatant (S) and membrane pellet (P) fractions of treated fly heads were subjected to Western blotting analyses, with Rh1 serving as a protein control for the membrane fraction (P). Quantification of the percentage of Gα_q_ protein in the cytoplasm is shown below. The complete genotypes are as follows: *w^1118^* (*wt*); *w^1118^*; *Gα_q_^V303D^* (*V303D*).

In addition to responding to light stimuli, *Drosophila* eyes have the ability to adapt to maintained illumination. Gα_q_ also participates in this long-term adaptation by shuttling between the cell membrane and the cytoplasm (Cronin *et al.* 2004; Frechter and Minke 2006). Under normal conditions, constant light stimulation results in the relocation of Gα_q_ to the cytoplasm, to prevent overactivation of the visual system (Kosloff *et al.* 2003). Upon termination of light stimulation, Gα_q_ returns to the membrane (Frechter *et al.* 2007). As shown in Figure 7, we were able to recapitulate this shuttling in wild-type eyes exposed to 2 hr of light stimulation. On the other hand, the V303D mutant protein was unable to relocate to the cytoplasm upon a similarly long exposure to light, suggesting that the *Gα_q_^V303D^* mutant is defective in long-term adaptation. However, we cannot rule out that the inability of the mutant protein to relocate is due to the lack of a photo response (*i.e.*, the light response is epistatic to the adaptation), even though we showed that the Rh1 receptor appears expressed and localized normally in the mutant (Figure 4).

## Discussion

In this study, we characterized a novel allele of the *Drosophila Gα_q_* gene. The mutant protein produced by this new allele possesses a single amino acid change from the wild-type version of an eye-specific isoform, yet it produces the strongest visual defects of all known *Gα_q_* mutants.

We suggest that the Gα_q_^V303D^ protein might have only affected the visual pathway at the level of the Gα_q_–PLC interaction. By Western blot and immunostaining analyses, we showed that key components of the phototransduction pathway are normal both at the protein level and at the subcellular localization level. In addition, the functional integrity of the remaining pathway is indicated by the normal kinetics of the residual light response in whole-cell recordings from mutant photoreceptors.

Structural studies suggest that the Val residue, which is mutated to Asp in our mutant, constitutes part of the interaction interface between Gα and its downstream effector. Interestingly, Val is replaced with Ile in Gα_q_ proteins from vertebrates, yet the hydrophobicity at this position is evolutionally conserved. Val and Ile appear interchangeable for *Drosophila* visual transduction, as the Gα_q_^V303I^ variant is functional under the conditions of our assays (Figure 2C). Therefore, it is highly likely that the change to a polar residue in V303D causes a major disruption of the interaction between Gα_q_ and its downstream effector, which in the case of *Drosophila* visual transduction is the PLC enzyme. The model that V303D loses its ability to activate PLC predicts that *Gα_q_^V303D^* would behave similarly to a *norpA* mutant with regard to the visual phenotypes. This is supported by existing data. First, *norpA* is one of very few mutants that produce a flat ERG response similarly to our *Gα_q_* mutant. Second, the *V303D* mutant phenocopies a *norpA* mutant in having the fastest rate of retinal degeneration induced by light.

Nevertheless, *Gα_q_^V303D^* behaves differently from a *norpA* mutant in one aspect. Gα_q_^V303D^ protein is defective in relocation to the membrane during prolonged exposure to lights, whereas Gα_q_ proteins in a *norpA* mutant behave normally (Kosloff *et al.* 2003; Cronin *et al.* 2004). Because the absence of PLC does not affect Gα_q_’s relocation behavior, the molecular defect in Gα_q_^V303D^ must also prevent its translocation to the cytoplasm upon constant light exposure. As shown by others (Kosloff *et al.* 2003; Cronin *et al.* 2004), this dynamic relocation to the cytoplasm appears to be affected only by the state of Rh1. Importantly, none of the downstream components of the signaling pathway is important to regulate the membrane-to-cytoplasm dynamics of Gα_q_, although the NinaC myosin III has a role in promoting the cytoplasm-to-membrane movement of Gα_q_ (Cronin *et al.* 2004). This would appear to imply that the *Gα_q_^V303D^* is also defective in its functional interaction with Rh1. However, our structural modeling suggests that this is unlikely to be the case. As shown in Figure 5, the V303D change might not have altered the overall structure of Gα_q_ including the regions important for GPCR interaction: helices 1 and 5. Therefore, the V303D mutant protein might be intrinsically defective in this membrane to cytoplasm shuttling. Further work is required to distinguish these possibilities.

In summary, we have recovered a new point mutation of the important Gα_q_ protein that essentially abolishes the visual transduction pathway in *Drosophila*. It also leads to one of the fastest rates of retinal degeneration induced by light. Although the molecular lesion lies in the interaction interface between Gα_q_ and its effector, functional characterization suggests that the mutant protein might harbor additional molecular defects. Therefore, our work reveals additional complexity in the regulation of G protein functions and generates a potential useful reagent for fine structural and functional studies of Gα_q_ in diverse organisms.
